# Heterogeneity in Kidney Histology and Its Clinical Indicators in Type 2 Diabetes Mellitus: A Retrospective Study

**DOI:** 10.3390/jcm12051778

**Published:** 2023-02-23

**Authors:** Shivendra Singh, Prem Shankar Patel, Archana Archana

**Affiliations:** 1 Department of Nephrology, Institute of Medical Sciences, Banaras Hindu University, Varanasi 221005, India; 2 Department of Nephrology, Indira Gandhi Institute of Medical Sciences, Patna 800014, India; 3 All India Institute of Medical Sciences, Patna 110029, India

**Keywords:** diabetes mellitus (DM), diabetic retinopathy (DR), diabetic nephropathy (DN), non-diabetic kidney disease (NDKD), kidney histology, clinical indicator

## Abstract

The heterogeneous spectrum of kidney disease in diabetes ranges from albuminuric or non-albuminuric diabetic kidney disease to non-diabetic kidney diseases. Presumptive clinical diagnosis of diabetic kidney disease may lead to an erroneous diagnosis. Material and Method: We analyzed the clinical profile and kidney biopsy of a total of 66 type 2 diabetes patients. Based on kidney histology, they were divided into—Class I (Diabetic Nephropathy), Class II (Non-diabetic kidney disease), and Class III (Mixed lesion). Demographic data, clinical presentation, and laboratory values were collected and analyzed. This study tried to examine the heterogeneity in kidney disease, its clinical indicator, and the role of kidney biopsy in the diagnosis of kidney disease in diabetes. Results: Class I consisted of 36(54.5%), class II 17(25.8%), and class III 13(19.7%) patients. The commonest clinical presentation was nephrotic syndrome 33(50%) followed by chronic kidney disease 16(24.4%) and asymptomatic urinary abnormality 8(12.1%). Diabetic retinopathy (DR) was present in 27(41%) cases. DR was significantly higher in the class I patients (*p* < 0.05). Specificity and positive predictive values of DR for DN were 0.83 and 0.81, respectively (sensitivity 0.61; negative predictive values 0.64). The Association of the duration of diabetes and the level of proteinuria with DN was statistically not significant (*p* > 0.05). Idiopathic MN (6) and Amyloidosis (2) were the most common isolated NDKD; whereas diffuse proliferative glomerulonephritis (DPGN) (7) was the commonest NDKD in mixed disease. Another common form of NDKD in mixed disease was Thrombotic Microangiopathy (2) and IgA nephropathy (2). NDKD was observed in 5(18.5%) cases in presence of DR. We noted biopsy-proven DN even in 14(35.9%) cases without DR, in 4(50%) cases with microalbuminuria and 14(38.9%) cases with a short duration of diabetes. Conclusion: Almost half (45%) of cases with atypical presentation have non-diabetic kidney disease (NDKD), though even among these cases with atypical presentation diabetic nephropathy (either alone or in mixed form) is commonly seen in 74.2% of cases. DN has been seen in a subset of cases without DR, with microalbuminuria, and with a short duration of diabetes. Clinical indicators were insensitive in distinguishing DN Vs NDKD. Hence, a kidney biopsy may be a potential tool for the accurate diagnosis of kidney disease.

## 1. Introduction

Traditionally, diabetic nephropathy (DN) is diagnosed clinically with evidence of proteinuria and diabetic retinopathy [1,2,3,4]. However, type 2 diabetes patients may develop various non-diabetic kidney diseases (NDKD) which are often missed on clinical grounds. The spectrum and prevalence of NDKD have been variably reported in different studies [5,6,7]. Remarkable heterogeneity in the spectrum and prevalence of NDKD has been demonstrated in a meta-analysis of a study involving kidney biopsy in diabetes [8]. Determining the clinical indicators suggestive of the type of nephropathy in diabetes is challenging. The sensitivity and specificity of these clinical indicators are variable. A recent study on kidney biopsy in diabetic patients has revealed the occurrence of DN or NDKD against their respective clinical indicators [5,7]. This evidence enforces kidney biopsy in diabetes as a gold standard tool for accurate diagnosis. However, the role of kidney biopsy in type 2 diabetes mellitus (DM) is debatable and remains to be elucidated [9,10]. Histological confirmation of DN or NDKD is important, particularly in the presence of atypical clinical presentation; because treatment and prognosis of NDKD are different. This study tried to examine the heterogeneity in kidney disease and its clinical indicator, and highlight the role of kidney biopsy in diagnosing kidney disease in diabetes mellitus.

## 2. Material and Method

This retrospective cohort study included type 2 diabetes mellitus patients, who underwent kidney biopsies between October 2016 to October 2022. The study included a total of 66 cases, whose complete clinical data were available for analysis. Patient of age > 18 years and both gender (male and female) was included in the analysis. Patients of age < 18 years and with proteinuria < 30 mg per day were excluded from the analysis. All patients’ demographic data and clinical presentations were reviewed and recorded from hospital records. Fundoscopic evidence of diabetic retinopathy was noted for all patients. Laboratory value of urine analysis, 24 h urinary protein, Complete Blood Count, renal function test, liver function test, lipid profile, immunological marker (RA factor, C3, C4, ANA, Anti ds DNA antibody, PR3 ANCA, MPO ANCA, and Anti GBM Ab), HBsAg, HCV, and HIV were obtained from records. Findings of light microscopy, immunofluorescence, and electron microscopy examination of kidney biopsy were reviewed and noted in detail for all patients. We analyzed the demographic data, clinical presentation, and laboratory value for the clinical syndrome, the indication of biopsy, and the type of nephropathy. Standard guidelines were used to define acute kidney injury (AKI), chronic kidney disease (CKD), and nephrotic syndrome [11,12]. 

Proteinuria was categorized into three categories: microalbuminuria (30–300 mg/day), Sub nephrotic (>300–3500 mg/day), and nephrotic range (>3500 mg/day) proteinuria. Based on kidney histology, patients were divided into three classes—Class I (Diabetic Nephropathy), Class II (Non-diabetic kidney disease), and Class III (Mixed lesion). Further analysis was done to find out the heterogeneity of kidney diseases, their clinical indicators, and the relevance of clinical indicators in the diagnosis of kidney disease in type 2 diabetes mellitus patients.

### Statistical Analysis

Statistical analysis was performed using PSPP version 1.4.1 (GNU Operating System, Free Software Foundation). Descriptive statistics were presented as the mean and SD for continuous, and number and percent for categorical variables. A one-way ANOVA variance analysis test was used to examine the significance of the difference between the three classes. Pearson’s chi-square test was used to compare the three classes for categorical variables. We used multinomial logistic regression to estimate odds ratios (ORs), associated 95% confidence intervals (95% CIs), and *p* values to know the association between the clinical indicators and kidney histology. We calculated the sensitivity, specificity, positive predictive value, and negative predictive value of the clinical indicators for DN. Observation considered statistically significant for *p*-value less than 0.05.

## 3. Result

**Figure 1:**
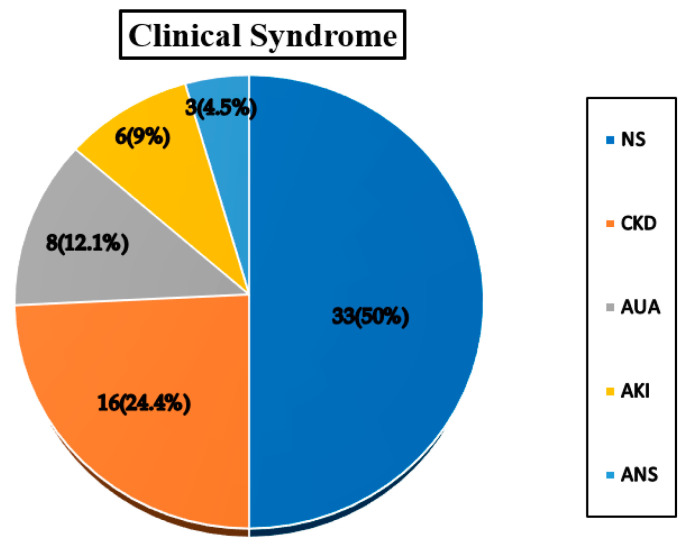
Presenting clinical syndrome in patients with type 2 diabetes mellitus (n = 66). NS: Nephrotic Syndrome, CKD: Chronic kidney Disease, AUA: Asymptomatic Urinary Abnormality, AKI: Acute Kidney Injury, ANS: Acute Nephritic Syndrome.

**Figure 2:**
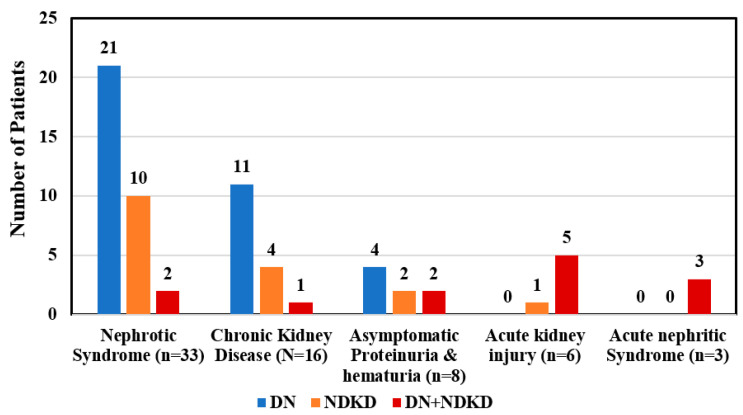
Heterogeneity of nephropathy in patients with type 2 diabetes patients with various clinical syndromes (n = 66).

A total of 66 patients (male 52; female 14) with male to female ratio of 3.7:1 was included. The mean age of patients was 51.1 ± 10.5 years. The mean serum creatinine was 2.7 ± 1.8 mg/dL. The average proteinuria of 63 patients was 3.8 ± 2.8 gm per day. Three patients were anuric. The mean duration of diabetes was 7.1 ± 3.9 years (Table 1). Based on kidney histology study, the population was grouped as Class I (Diabetic Nephropathy), Class II (Non-diabetic kidney disease), and Class III (Mixed lesion). Class I consisted of 36(54.5%), class II 17(25.8%), and class III 13(19.7%) patients. Class-wise patient characteristics are mentioned in the table, and the three classes did not have statistically significant differences concerning age, sex, serum creatinine, proteinuria, and duration of diabetes (*p* > 0.05) (Table 2). The presenting clinical syndrome and an indication of kidney biopsy were: nephrotic syndrome 33(50%), chronic kidney disease 16(24.2%), asymptomatic proteinuria and hematuria 8(12.1%), acute kidney injury 6(9.1%), and acute nephritic syndrome 3(4.5%) (Figure 1). Diabetic retinopathy (DR) was found in 27(40.9%). Isolated DN was seen in 36(54.5%) cases; remaining 30(45.5%) cases had NDKD either in isolation 17(25.8%) or in mixed 13(19.7%) form. Patients with nephrotic syndrome (n = 33) had isolated DN in 21(63.6%), isolated NDKD in 10(30.3%), and mixed disease in 2(6.1%) cases. In patients with chronic kidney disease; 11(68.7%) had isolated DN, and the remaining 4(25%) cases had isolated NDKD and one mixed lesion. Four (50%) patients with asymptomatic urinary abnormalities had isolated DN; the remaining had isolated NDKD in 2(25%) and NDKD mixed with DN in 2(25%) cases. NDKD was the most common lesion in patients presenting with acute kidney injury in 6(isolated NDKD 1, mixed lesion 5) and acute nephritic syndrome in 3(100%) (Figure 2). Idiopathic MN (6) and Amyloidosis (2) were the most common isolated NDKD. Membranoproliferative glomerulonephritis, Lupus nephritis, diffuse proliferative GN, Mesangioproliferative GN, Hypertensive Nephropathy, Xanthogranulomatous pyelonephritis, Thrombotic Microangiopathy (TMA), Chronic tubulointerstitial nephritis (CTIN), and Light chain deposition disease (LCDD) were another NDKD each in one case. The commonest NDKD in the mixed lesion were DPGN (7), followed by TMA (2), IgA nephropathy (2), pauciimune GN (1), and ANCA negative renal limited vasculitis (1) (Table 3). Of 8 cases with microalbuminuria, 4(50%) had Diabetic Nephropathy (isolated DN 3; mixed 1) and in remaining 4(50%) patients had isolated NDKD. In 37 cases with nephrotic range proteinuria; the majority 24(64.9%) had isolated DN. However, the remaining 10(27%) cases had isolated NDKD, and in 3(8.1%) cases mixed lesions. Duration of diabetes was <5 years in 32(48.5%), between 5–10 years in 19(28.8%), >10 years in 15(22.7%) cases. Isolated DN was seen in 14(43.8%) patients with diabetes of <5 years; while the remaining cases had isolated NDKD and NDKD mixed with DN in 11(34.3%) and 7(21.9%) cases, respectively. Predominantly isolated DN was seen in 11(73.3%) patients with diabetes of >10 years. However, NDKD either alone or in mixed form was noted in the remaining 4(26.7%) cases even with diabetes of >10 years. DR was significantly higher in the class I patients (*p* < 0.05). Isolated DN was seen in the majority of 22(81.5%) patients with diabetic retinopathy. However, NDKD either alone or in mixed form was noted in the remaining 5(18.5%) cases even in presence of DR. Isolated DN was noted in 14(35.9%) patients without DR. DN was the predominant lesion in presence of DR (81.5%), while NDKD either alone or in mixed form was a predominant lesion in the majority (64%) in the absence of DR. Sensitivity, specificity, negative and positive predictive value of DR for diabetic nephropathy are mentioned in the Table 4. The association between clinical indicators and non-diabetic kidney disease is shown in Table 5.

## 4. Discussion

In the present study, we retrospectively reviewed the demographic data, clinical features, and kidney biopsy of a total of 66 type 2 diabetes patients. The number of participants in the present analysis is low, which could be a reflection of a lack of awareness, resource limitation, limited access to a tertiary health care facility, and a reluctant strategy for a kidney biopsy at this center. This limits the power of the study and restricts the generalization of results. We found the mean age of the patient was 51.1 ± 10.5 years, and the female patient was 14(21%). The mean duration of diabetes in our cohort was 7.1 ± 3.9 years. The average amount of proteinuria was 3.8 ± 2.8 gm per day. The mean serum creatinine was 2.7 ± 1.8 mg/dL. The mean age and mean duration of diabetes in Class II (isolated NDKD) were comparatively lower than Class I (DN) and Class III (DN + NDKD). However, the level of 24-h proteinuria in Class II (4.2 ± 3.3 gm) was comparatively higher than in Class I (4.0 ± 2.3 gm) and Class III (2.8 ± 3.2 gm). The mean serum creatinine in Class III was higher than in class I and Class II (3.8 vs. 2.4 vs. 2.6 mg/dL), reflecting either advanced or acute worsening of the disease. The three classes did not have statistically significant differences concerning age, sex, serum creatinine, proteinuria, and duration of diabetes (*p* > 0.05). Our findings regarding mean age, duration of diabetes, and 24 h urine protein excretions were like other studies [13,14,15,16]. Most common presentation was nephrotic syndrome in 33(50%) patients. Remaining patients presented with chronic kidney disease 16(24.2%), asymptomatic urinary abnormality 8(12.1%), acute kidney injury 6(9.1%) and acute nephritic syndrome 3(4.5%). Similarly, other studies also reported the heterogeneous presentation of kidney disease in diabetes [16,17]. DN predominantly presents with either nephrotic syndrome or chronic kidney disease; while NDKD tends to present predominantly with acute kidney injury or acute nephritic syndrome [18]. Indications of kidney biopsy in diabetic patients have not been specified and remain to be elucidated. Policies of kidney biopsy in diabetes vary from center to center, and largely depend on individual factors, clinician decisions, and clinically indicated [8,10,19]. Research indicated kidney biopsy was performed in only a few studies [20]. Kidney biopsy is often considered whenever clinical course the is atypical and there is a strong suspicion of non-diabetic kidney disease. A total of 66 type 2 diabetes patients underwent kidney biopsy for atypical presentations with clinical indications during the study period. Nephrotic syndrome was the commonest indication of kidney biopsy in 33(50%) cases in the present series, followed by Chronic kidney disease 16(24.4%). At our center in routine clinical practice, we do not perform a kidney biopsy in asymptomatic type 2 diabetes patients without clinical indications, and with a typical clinical course. These could be indications of kidney biopsy for research purposes. Thus, this could be a selection bias in the present study. Our study demonstrated isolated DN in 36(54.5%) cases. The reported prevalence of isolated DN varies from 6.5% to 94% in patients with type 2 diabetes in various studies from across the world [8,19,20]. Classical way of diagnosis of diabetic kidney disease (DKD) is the appearance of progressive albuminuria with or without reduction in glomerular filtration rate (GFR) [2]. However, many times such a paradigm is not followed. In the last few decades, this idea has been changed and emerging evidence suggests a more diverse presentation of DKD, which is not consistent with the classical paradigm [3,4,21]. Recently several retrospective studies of kidney biopsy in diabetes have shown histological evidence of DKD in patients with normoalbuminuria [8,21,22]. About 20% of patients with type 2 diabetes and 25% with type 1 diabetes develops biopsy-proven DN without albuminuria (non-albuminuric DKD) [3,4]. Thus, the difference in diagnostic criteria of DKD and the threshold of kidney biopsy in diabetes could be the reason behind the variation in the prevalence of DKD in different studies. The prevalence of NDKD either alone or superimposed on DN varies and ranges from 3% to 82.9% of the total kidney biopsies [8]. We found isolated NDKD in 17(25.8%), and NDKD mixed with DN in 13(19.7%) cases. Among isolated NDKD, idiopathic MN was the most common lesion in six and followed by Amyloidosis in two patients. The commonest NDKD in the mixed lesion were DPGN (7), followed by TMA (2), IgA nephropathy (2), pauciimune GN (1), and renal limited vasculitis (1). In a meta-analysis by Fiorentino et al., the most commonly reported NDKD were IgA nephropathy (IgAN) followed by Membranous nephropathy (MN), focal segmental glomerulosclerosis (FSGS), and tubulointerstitial nephritis (TIN) [8]. In an Indian study, the author reported membranous nephropathy (MN) as the commonest NDKD in 12.9% of type 2 diabetic patients [13]. The prevalence of membranous nephropathy in patients with diabetes is variable and ranges from 11.9% [23] to 30% [24]. Thus, our observations also demonstrated the diversity in kidney disease in type 2 diabetes patients as in other studies [5,6,7,8,18]. Prevalence of different types of NDKD was different in the different geographical regions. FSGS is commonly seen in patients from Europe and United State; compared to IgAN in Asia [8,25]. In a study in the United States including 620 patients, the most commonly seen NDKD was (FSGS) [25]. Minimal change disease (MCD) was also reported as NDKD in diabetes [20]. This wide difference in the frequency and spectrum of NDKD in various studies could be due to diversity in the kidney biopsy policy, and regional and/or racial variations of the study cohort. It is important to identify the clinical indicators helpful in the clinical diagnosis of DN vs. NDKD, in performing the kidney biopsy to make a correct diagnosis. Multiple clinical factors like the duration of diabetes, features of DR, and level of proteinuria are used to differentiate DN from NDKD [13,14,15,16,17,18,19,20]. Classically long duration of diabetes (>10 years), presence of DR, and severe proteinuria strongly suggest DKD [25,26,27]. Whereas NDKD either isolated or mixed was more common (56% vs. 44%) than DN in patients with diabetes of <5 years. However, this dictum is not always followed. The majority 11(73%) of our patients with diabetes of more than 10 years had isolated DN and approximately 27% cases had non-diabetic kidney disease despite long duration of diabetes. Similarly, Prakash et al., also reported DN as predominant lesion in patients with diabetes duration > 10 years and NDKD as predominant lesion in patients with diabetes duration < 5 years [13]. Thus, our observation supports that a longer duration of diabetes is strongly associated with DN found in other studies [19,24]. Although albuminuria is considered a clinical hallmark of DKD and the prevalence of DKD increases with the degree of proteinuria. The reverse is not true. However, recent evidence has shown that a significant number of diabetes patients had non-albuminuric DKD. Kidney histology in diabetic patients with normoalbuminuria revealed histological features of the advanced diabetic glomerular lesion, and histological changes were diverse in nature [3,4,21,22]. As we earlier discussed regarding the policy of kidney biopsy in diabetes at our center, the present study did not include non-albuminuric patients. However, now a day this subgroup represents significant proportions of diabetic individuals with reduced GFR [3,4]. Thus, this is another limitation of the present study. We observed Diabetic Nephropathy in 4(50%) (isolated DN 3; mixed 1) cases with microalbuminuria, and the remaining 4(50%) patients had isolated NDKD. Our study also demonstrated the increase in prevalence of DN with increase in level of proteinuria, but a subset of patients with sub-nephrotic (50%) or nephrotic range proteinuria (35%) had biopsy proven NDKD. Patients with a duration of diabetes of fewer than 5 years (odds ratio 4.97; 95% CI, 0.49–50.58; *p* = 0.175) and microalbuminuria (odds ratio 2.03; 95% CI, 0.28–14.46; *p* = 0.479) had a high risk of NDKD, but it was not statistically significant. Hence, recent data do not support the classical paradigm of diabetic kidney disease. Thus, our observation reasonably suggests that the level of proteinuria does not discriminate between DN and NDKD, and proteinuria is a poor predictor of the type of nephropathy in type 2 diabetes. The prevalence of diabetic retinopathy vary widely and ranges from 30–60% [13,14]. In the present study diabetic retinopathy was noted in 27(40.9%) cases. The Association of diabetic retinopathy with DKD is well established, although the strength of the association is variably reported [13,16,20,27,28]. A recent meta-analysis revealed that the sensitivity and specificity of DR in predicting DN were only 65% (95% CI 0.62–0.68) and 75% (95% CI 0.73–0.78), respectively [28]. However, in another study Tone et al., observed that evidence of DR had the highest sensitivity (87%) and specificity (93%) for DKD. This observation of the absence of DR as a strong indicator of NDKD was also supported by another study [27]. However, recent evidence does not agree with this concept that the mere absence of DR excludes the possibility of NDKD; because various studies had shown a high proportion (50–70%) of DN cases did not have diabetic retinopathy [16,19,20,28]. Prakash et al., have reported DKD in 25–43% of cases without DR [16]. The prevalence of DR was significantly higher in the class I patients (*p* < 0.05). Isolated DN was seen in the majority of 22(81.5%) patients with diabetic retinopathy. Although, evidence of DR strongly suggest diagnosis of DKD but does not exclude the possibility of NDKD. Our findings also support the afore statement, because we noted biopsy proven NDKD (either alone or mixed form) in the remaining 5(18.5%) cases even in presence of DR. Isolated DN was noted in 14(35.9%) patients in absence of DR, while the remaining 25 patients had isolated NDKD in 15(38.5%) and NDKD mixed with DN in 10(25.6%). DN was the predominant lesion in presence of DR (81.5%), while NDKD either alone or in mixed form was a predominant lesion in the majority (64%) in the absence of DR. Although, specificity and positive predictive values of DR for DN were high (0.83 and 0.81, respectively); it had low sensitivity (0.61) and negative predictive values (0.64). We demonstrated that the absence of diabetic retinopathy was strongly associated with the presence of NDKD (odds ratio 9.61; 95% CI, 1.79–51.45; *p* = 0.008). Our finding also backs the observation of other published studies [19,20,28]. Thus, diabetic retinopathy is a poor predictor of the type of nephropathy in type 2 diabetes.

To summarize, our observation as well as the other published report reasonably suggest the heterogenous nature of kidney disease in type 2 diabetes. Emerging evidence had demonstrated that DKD may occur in a patient with a short duration of diabetes, with normo or microalbuminuria, and in absence of diabetic retinopathy. Thus, clinical indicators are a poor predictor of the type of nephropathy in type 2 diabetes and may lead to an erroneous diagnosis of DKD. Currently, there is no consensus on performing kidney biopsies in diabetes. But evidence enforcing kidney biopsy as the gold standard tool for early and accurate diagnosis of kidney disease in diabetes, many of them are treatable and reversible.

## 5. Conclusions

Almost half (45%) of cases with atypical presentation have non-diabetic kidney disease (NDKD), though even among these cases with atypical presentation diabetic nephropathy (either alone or in mixed form) is commonly seen in 74.2% of cases. About one-third (36%) of cases without DR have diabetic nephropathy, and about 20% of cases with DR have non-diabetic kidney disease on kidney biopsy. An almost equal number of cases have Diabetic nephropathy and NDKD in the presence of short diabetes duration. Factors like duration of diabetes, level of proteinuria, and presence of DR are not sensitive indicators for distinguishing DN vs. NDKD. Thus, clinical diagnosis alone may give an erroneous diagnosis. Hence, a kidney biopsy may be a potential tool for the early and accurate diagnosis of heterogeneous kidney disease in diabetes mellitus.

### Limitation of Study

The major limitation of this study was the small number of participants, participants with symptomatic disease, and Kidney biopsy in advanced disease.

## Figures and Tables

**Table 1:** The clinical characteristics of patients with type 2 diabetes mellitus (n = 66).

Characteristics	Results
Age (Mean ± SD) years	51.1 ± 10.5
Male: Female	52:14
Serum Creatinine (Mean ± SD) mg/dL	2.7 ± 1.8
24-h urinary protein (Mean ± SD) gm/day	3.8 ± 2.8
Duration Of Diabetes Mellitus (Mean ± SD) years	7.14 ± 3.9
Diabetic retinopathy n (%)	27(40.9)

**Table 2:** Class-wise clinical characteristics of patients with type 2 diabetes mellitus (n = 66).

Characteristics		Class I (DN) 36(54.5%)	Class II (NDKD) 17(25.8%)	Class III (DN + NDKD) 13 (19.7%)	*p*-Value
Age (Mean ± SD) years		51.3 ± 9.4	48.2 ± 9.6	54.5 ± 13.7	0.266(NS)
Male: Female ratio		27:9	13:4	12:1	0.410(NS)
Serum Creatinine (Mean ± SD) mg/dL		2.4 ± 1.5	2.6 ± 1.8	3.8 ± 2.5	0.070(NS)
24-h urinary protein (Mean ± SD) gm/day		4.07 ± 2.3	4.20 ± 3.3	2.80 ± 3.2	0.321(NS)
Duration Of Diabetes Mellitus	<5 years (n = 32)	14	11	7	0.291(NS)
	5–10 years (n = 19)	11	5	3	
	>10 years (n = 15)	11	1	3	
Diabetic retinopathy	Present (n = 27)	22	2	3	0.001(S)
	Absent (n = 39)	14	15	10	
Level of Proteinuria	Microalbuminuria (n = 8)	3	4	1	0.003(S)
	Sub-nephrotic proteinuria (n = 18)	9	3	6	
	Nephrotic Proteinuria (n = 37)	24	10	3	

NS—Not Significant, S—Significant, DN—Diabetic Nephropathy, NDKD—Non Diabetic Kidney Disease.

**Table 3:** Heterogeneity of NDKD in patients with type 2 diabetes mellitus patients (n = 30).

Type of Isolated NDKD	n (Number)
(a) Membranous nephropathy (MN)	6
(b) Amyloidosis	2
(c) Membranoproliferative GN (MPGN)	1
(d) Lupus nephritis (LN)	1
(e) Diffuse proliferative GN (DPGN)	1
(f) Mesangioproliferative GN	1
(g) Hypertensive Nephropathy	1
(h) Xanthogranulomatous pyelonephritis	1
(i) Chronic tubulointerstitial nephritis (CTIN)	1
(j) Thrombotic Microangiopathy (TMA)	1
(k) Light chain deposition disease (LCDD)	1
**Type of NDKD in Mixed**	**n (Number)**
(a) DN+ Diffuse proliferative GN (DPGN)	7
(b) DN + Thrombotic Microangiopathy (TMA)	2
(c) DN + IgA nephropathy	2
(d) DN + pauciimune GN	1
(e) DN + Renal limited vasculitis	1

DN: Diabetic Nephropathy, GN: Glomerulonephritis, NDKD: Non-Diabetic Kidney Disease.

**Table 4:** Crosstabulation shows the association between clinical indicators and diabetic nephropathy (n = 66).

Variable	Sensitivity	Specificity	Positive Predictive Value	Negative Predictive Value
Diabetes Duration > 10 years	0.30	0.86	0.73	0.50
Proteinuria > 3.5 g/day	0.66	0.51	0.64	0.53
Diabetic Retinopathy	0.61	0.83	0.81	0.64

**Table 5:** Multinomial logistic regression shows the association between clinical indicators and non-diabetic kidney disease.

Variables	Odds Ratio (OR)	95% CI (Confidence Interval)	*p* Value
Diabetes duration < 5 years	4.97	0.49–50.58	0.175
Microalbuminuria	2.03	0.28–14.46	0.479
Absence of Diabetic Retinopathy	9.61	1.79–51.45	0.008

## Data Availability

The data presented in this study are available on request from the corresponding author. The data are not publicly available due to privacy.
